# Anaesthesia in a patient with subarachanoidal haemorrhage and high oxygen affinity haemoglobinopathy (HB york): case report

**DOI:** 10.1186/1471-2253-12-19

**Published:** 2012-08-08

**Authors:** Enrico Monaca, Tobias Jüttner, Norbert Gattermann, Michael Winterhalter

**Affiliations:** 1 Department of Anaesthesiology, Heinrich-Heine-University, Moorenstrasse 5, D-40225, Düsseldorf, Germany; 2 Department Of Haematology, Oncology and Clinical Immunology, Heinrich-Heine-University, Moorenstrasse 5, D-40225, Düsseldorf, Germany

**Keywords:** Haemoglobinopathy, Hb York, Oxygen affinity, Neuroanaesthesia, Subarachnoid haemorrhage, Anaemia, Hypoxia

## Abstract

**Background:**

Approximately 90 haemoglobinopathies have been identified that result in abnormally high oxygen affinity. One of these is haemoglobinopathy York (HbY), first described in 1976. HbY causes an extreme leftward shift of the oxygen dissociation curve with the P50 value changing to 12.5 - 15.5 mmHg (normal value 26.7 mmHg), indicating that approximately half of the haemoglobin is not available as oxygen carrier. Patients with haemoglobinopathies with increased oxygen affinity could suffer from the risk developing ischaemic complications due to a lack of functional oxygen carriers. This is, to best of our knowledge, the first case report on a patient with HbY published in connection with anesthesia.

**Case Presentation:**

A 42-year-old female with a severe headache and Glasgow coma scale (GCS) of 15 was admitted to the neurosurgical intensive care unit with a ruptured, right sided ICA aneurysm with consecutive subarachnoid haemorrhage [Fisher III, World Federation of Neurosurgical Societies (WFNS) I)]. The medical history of the patient included an erythrocytosis (Hb 17.5 g/dl) on the base of a high-oxygen-affinity haemoglobinopathy, called Hb York (HbY). With no time available to take special preoperative precautions, rapid blood loss occurred during the first attempt to clip the aneurysm. General transfusion procedures, according to the guidelines based on haemoglobin and haematocrit values, could not be applied due to the uncertainty in the oxygen carrier reduction. To maintain tissue oxygen supply, clinical indicators of ischaemia were instead utilized to gauge the appropriate required blood products, crystalloids and colloids replacements. Despite this, the patient survived the neurosurgical intervention without any neurological deficit.

**Conclusions:**

Family members of patients with HbY (and other haemoglobinopathies with increased oxygen affinity) should undergo clinical assessment, particularly if they are polycythaemic. If the diagnosis of HbY is confirmed, they should carry an "emergency anaesthesiology card" in order to avert perioperative risks arising from their "hidden" anemia.

## Background

Haemoglobinopathy is defined as a genetic defect that results in an abnormal structure of one of the globin chains of the haemoglobin molecule. It is the most frequently occurring monogenic disorder [1]. To date, more than 1000 varieties have been described. Although haemoglobinopathies often entail abnormal laboratory findings like erythrocytosis and/or haemolysis, only a few cause severe clinical symptoms. Well-known examples of haemoglobinopathies with severe clinical phenotype are sickle cell disease, beta-thalassemia or Hb S/C disease.

Approximately 90 haemoglobinopathies have been identified that result in abnormally high oxygen affinities. The first was reported in 1966 on an 81-year-old patient who presented with polycythemia [2]. Decreased oxygen supply causes erythrocytosis, with clinical features depending on the degree of abnormal oxygen affinity. This subgroup of haemoglobinopathies includes haemoglobinopathy York (HbY), first described in 1976 [3]. A second and third case was reported in 1983 and 2001, respectively [4,5]. Patients with haemoglobinopathies with increased oxygen affinity could suffer from an additional increased risk developing ischaemic complications when undergoing surgery with a higher risk of blood loss or circulatory arrest due to a lack of functional oxygen carriers. In 1997, an 11-year-old patient with Hb Bryn Mawr was reported who was intensively prepared for a cholecystectomy by performing partial exchange transfusion to prevent tissue hypoxia during anaesthesia. Hb Bryn Mawr is an unstable Hb variant resulting in congenital haemolytic anaemia and increased oxygen affinity [6]. We wish to report the case of a patient with HbY who survived a life-threatening situation characterized by subarachnoid haemorrhage and severe perioperative aneurysmal bleeding. To the best of our knowledge, this is only the fourth published case of HbY worldwide and the first case published in connection with anaesthesia.

## Case presentation

A 42-year-old female with a severe headache and Glasgow Coma Scale (GCS) of 15 was admitted from a regional hospital close to our neurosurgical intensive care unit. Digital subtraction angiography showed a ruptured, right-sided ICA aneurysm near the bifurcation followed by a subarachnoid haemorrhage (Fisher III, World Federation of Neurosurgical Societies I: GCS 15, no motoric deficit). A neurological examination revealed no deficits. The medical history of the patient included obesity (a body mass index of 29), Hashimoto thyroiditis, and an erythrocytosis (Hb 17.5 g/dl), in addition to the HbY. The diagnosis of HbY was initially performed at the University of Ulm (Germany) in 2002 through molecular genetic analysis, subsequent to the exclusion of alternative explanations for erythrocytosis. Preoperative laboratory findings showed a haematocrit of 54%, Hb 17.5 g/dl, reticulocytes 25/1000 and erythrocytes 6.2 x 10^6^/μl.

After induction of anaesthesia with propofol (2 mg/kg), remifentanyl (0.5 μg/kg/min) and rocuronium (70 mg), an arterial and a central-venous catheter were placed in addition to two peripheral intravenous 17 G catheters. The lungs were ventilated with a fraction of inspired oxygen (FiO_2_) of 0.5 and a minute ventilation (MV) of 110 ml/min/kg. Nimodipine was continually administered at a rate of 1 mg/h. Anaesthesia was maintained with Propofol (8 mg/kg/h) and Remifentanyl (0.25 μg/kg/h). The operation was started and progressed uneventfully until the surgeon tried to dissect the basis of the aneurysm, which led to severe bleeding. Several temporary clips were required to stop the haemorrhage (approx. 1600 ml). Subsequent to volume resuscitation with 1000 ml Ringer's solution and 500 ml of volume expander (hydroxyethyl starch; HAES) 130/0.4, the haemoglobin value was 10.8 g/dl. During the bleeding interval, no S-T segment changes were observed. However, typical signs of rapid fluid loss, such as a moderately increased heart rate and a temporary drop in blood pressure, were present. Systolic blood pressure was always kept between 120 mmHg and 140 mmHg using noradrenalin in varying dosages (max. 0.25 μg/kg/min). Meanwhile, the patient received 6 units of packed red blood cells (PRBC) and further fluid substitutions.

Further bleeding was stopped by continuous compression with TachoSil®, enabling the obliteration of the aneurysm. No vascular occlusions were detected on an indocyanine green angiography. The blood pressure was subsequently pharmacologically raised to a systolic value of approximately 140 mmHg, with no further detectable haemorrhaging. It was possible to extubate the patient within a few minutes upon completion of the operation. Immediate neurological assessment revealed no detectable deficits. The patient was transferred to the intensive care unit with haemoglobin of 16.8 g/dl. Further recovery was uneventful.

## Conclusions

This case report describes a high urgency neurosurgical intervention in a patient with HbY. There was no time for taking special preoperative precautions, as described by Larson *et al*. [6], as our patient experienced sudden and rapid blood loss. The first Hb value was 10.8 g/dl. The guidelines of the German Medical Association recommend that attempts should be made to maintain intraoperative Hb concentrations above 10 g/dl in case of acute bleeding. Taking into account the fact that approximately half of the patient's haemoglobin was non-functional, a more accurate estimate of the "functional" or "actual" Hb level would have been about 5.4 g/dl. Fortunately, the reaction to the sudden blood loss was not dramatic. There was neither clinical nor laboratory evidence of ischemia. Our patient was generously transfused, achieving a haemoglobin concentration of 16.8 g/dl by the end of surgery. However, it appears rather difficult to assess how much haemoglobin was functional when a blood loss of 1600 ml (containing approximately 800 ml HbY) was substituted with 1200 ml "healthy" PRBC with a haematocrit of 60%. When performing such a rough or ‘ballpark’ estimate, the use of the following calculation is proposed: Starting with a Hb of 17.5 g/dl, a blood loss of approximately 40% to 10.8 g/dl was nearly compensated with PRBC to Hb 16.8 g/dl. The lost blood contained HbY and "healthy" blood in an equal ratio, thus 20% of lost blood was "pure" HbY. This was substituted with 100% healthy blood, leading to a shift in the HbY:"healthy" blood ratio of 30:70 (50%-20%/50% + 20%) and an estimated value of a functional haemoglobin concentration of 11.8 g/dl (70% of 16.8 g/dl). However, a storage-related depletion of 2.3-diphosphoglycerate (2.3-DPG) content in erythrocytes causing an additional leftward shift of the oxygen-dissociation curve could decrease oxygen transport capacity.

During anaesthesia, we discover no deviation in pulse oxymetry readings in comparison to arterial blood gas analysis. This fact stands in apparent contrast to a theoretical overestimation of pulse oxymetric values, as half of the haemoglobin is already- and remains irreversible saturated with oxygen. For example, overestimated values measured with commonly used two-wave pulse oxymeters are found in patients with carbon monoxide intoxication due to the similar absorption spectrum of irreversible saturated haemoglobin and oxygenated haemoglobin. In addition overestimated oxymetry readings have also been found in, for example, high oxygen affinity haemoglobin Koln, which is associated with a left-shifted oxygen dissociation curve [7]. Taking into account the limitations of conventional oximetry, especially in these patient populations, an alternative monitoring method should be employed, such as arterial blood gas analysis or end-tidal oxygen concentration monitoring [8,9]. Furthermore, new presence of arrhythmia or changes of S-T segment, an increase of oxygen extraction rate greater than 50% and a decrease of oxygen consumption of more than 10% could serve as commonly accepted assessment of an adequate oxygen delivery.

The overall goal was to maintain blood pressure at a preoperative level until the aneurysm was taken care of. This was achieved by adjusting accordingly the levels of noradrenalin administration. During the acute bleeding, the preferred technique for gaining control over the ruptured aneurysm was to temporarily occlude the vessel instead of a transiently decreasing the mean arterial pressure. As part of the triple-H therapeutic strategy, haemodilution could not be performed as the team identified hypoxia as the most dangerous threat to the patient and tried to stabilize the "actual" Hb at a reasonably high level. The choice of anaesthetic drugs led to a rapid and smooth emergence from anaesthesia, which allowed an early neurological assessment.

The patient's history of haemoglobinopathy begun when a general practitioner suspected polycythaemia vera as an underlying cause for a chronic erythrocytosis. Common causes, including cigarette smoking, psychosocial stress, chronic residence at high altitudes and chronic lung disease, were excluded. Finally, HbY (α_2_ β_2_^146Pro^) was identified by DNA sequencing, which indicated an autosomal dominant inherited point mutation of codon 146, where adenosine was exchanged for cytosine (CAC ⇒ CCC). The mutation causes a change from histidine to proline at position 146 in the β-globin chain. The mutant globin has approximately twice the normal affinity for oxygen [3], causing an extreme leftward shift of the oxygen dissociation curve, with the P50 changing from 26.5 ± 1.0 mmHg to approximately 12.5 - 15.5 mmHg [4,5,10]. To date, only heterozygotes carriers have been detected, suggesting that this mutation is lethal for homozygotes. In heterozygote individuals the red blood cells contain a 1:1 mixture of HbY and normal HbA. The mutant HbY always remains saturated with O_2_ and thus cannot serve the physiological function of oxygen delivery. As a consequence of decreased tissue oxygen supply, erythropoietin production in the kidneys is upregulated, resulting in erythrocytosis. Preoperative laboratory findings confirmed an usual compensatory mechanism for chronic but moderate tissue hypoxia.

Young individuals with Hb York are usually asymptomatic. However, they are permanently at risk to suffer from a state of "functional anaemia". Therefore, the following general advice may be justified: insufficient tissue perfusion should be avoided by generous fluid intake, especially during hot weather or when pyrexia or diarrhoea occurs; all pulmonary infections should be treated without delay; and patients should not smoke and should avoid travelling to high-altitude locations. Until more knowledge has accumulated, it is difficult to comment on the prognosis of individuals with HbY. As yet, these individuals seem to have a normal life expectancy. Nevertheless, they may become symptomatic when their ability to compensate for their 50% dysfunctional haemoglobin is compromised by congestive heart failure, coronary heart disease, cerebrovascular insufficiency, peripheral arterial disease, chronic or acute lung disease, or pregnancy. In addition, carbon monoxide intoxication or intoxications causing methaemoglobinemia or sulfhaemoglobinemia may lead to critical situations due to a critical level of dysfunctional haemoglobin. The same may be true for rapid blood loss.

Chronic compensatory changes in patients suffering from quantitative and qualitative anaemic diseases, include an up regulated cardiac output, a decreased vascular resistance, increased amount of erythrocyte and a higher content of 2.3-DPG, which causes a right-shift and thus a minimal compensation of the extremely left shifted oxygen dissociation curve. Erythrocytosis causing increased blood viscosity can lead to thromboembolic complications, especially in elderly individuals with haemoglobinopathy [11]. Recent investigations suggest that preoperative anaemia increases the risk of postoperative mortality [12]. In our patient, the preoperative situation was characterized by a "functional" anaemia, a lack of an appropriate level of oxygen carriers, despite a Hb concentration of 17.5 g/dl. Severe intraoperative haemorrhaging aggravated the situation. Fortunately, the patient tolerated this complication rather well with the immediate volume substitution, catecholamine administration and a generous blood transfusion. We feel that our experience does not allow us to draw any conclusions as to the benefit of partial exchange transfusions as a preoperative preparatory regimen. Additionally, the special precautions carried out by Larson *et al*. in 1997 in a patient with Hb Bryn Mawr seemed to be an excessive precaution [6]. From our point of view, a young person who lived his entire life in complete stability and most probably in adequate oxygen delivery environment does not need an exchange transfusion for a minor procedure.

Family members of patients with HbY (and other haemoglobinopathies with increased oxygen affinity) should undergo clinical assessment, particularly if they are polycythaemic. If the diagnosis of HbY is confirmed, they should carry an "emergency anaesthesiology card" in order to avert perioperative risks arising from their "hidden" anaemia. It should be emphasized that general transfusion guidelines are not applicable to individuals with this rare condition. Therapeutic attempts at decreasing the abnormal oxygen affinity using bezafibrates are still being investigated. Venesection seems to only be sensible in case of repetitive thrombotic events, but the risk of an ischaemia is always present in these patients.

## Consent

Written informed consent was obtained from the patient for publication of this case report. A copy of the written consent is available for review by the Editor-in-Chief of this journal.

## Competing Interests

The authors declare that they have no competing interests.

## Authors’ contributions

EM: Preparation of the manuscript and involvement in the case. TJ: Anaesthesiologist involved in the case. NG: Substantial hematological background and preparation of the manuscript. MW: Preparation of the manuscript. All authors read and approved the final manuscript.

## Pre-publication history

The pre-publication history for this paper can be accessed here:

http://www.biomedcentral.com/1471-2253/12/19/prepub
